# RNF4 and USP7 cooperate in ubiquitin-regulated steps of DNA replication

**DOI:** 10.1098/rsob.230068

**Published:** 2023-08-23

**Authors:** Ya-Chu Chang, Kevin Lin, Ryan M. Baxley, Wesley Durrett, Liangjun Wang, Olivera Stojkova, Maximilian Billmann, Henry Ward, Chad L. Myers, Anja-Katrin Bielinsky

**Affiliations:** ^1^ Department of Biochemistry, Molecular Biology and Biophysics, University of Minnesota, Minneapolis, MN 55455, USA; ^2^ Department of Computer Science and Engineering, University of Minnesota, Minneapolis, MN 55455, USA

**Keywords:** STUbL, SUMO, ubiquitin, genome stability, RNF4, USP7

## Abstract

DNA replication requires precise regulation achieved through post-translational modifications, including ubiquitination and SUMOylation. These modifications are linked by the SUMO-targeted E3 ubiquitin ligases (STUbLs). Ring finger protein 4 (RNF4), one of only two mammalian STUbLs, participates in double-strand break repair and resolving DNA–protein cross-links. However, its role in DNA replication has been poorly understood. Using CRISPR/Cas9 genetic screens, we discovered an unexpected dependency of *RNF4* mutants on *ubiquitin specific peptidase 7* (*USP7)* for survival in *TP53*-null retinal pigment epithelial cells. *TP53^−/–^/RNF4^−/–^/USP7^−/–^* triple knockout (TKO) cells displayed defects in DNA replication that cause genomic instability. These defects were exacerbated by the proteasome inhibitor bortezomib, which limited the nuclear ubiquitin pool. A shortage of free ubiquitin suppressed the ataxia telangiectasia and Rad3-related (ATR)-mediated checkpoint response, leading to increased cell death. In conclusion, RNF4 and USP7 work cooperatively to sustain a functional level of nuclear ubiquitin to maintain the integrity of the genome.

## Graphical abstract

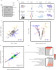

## 1. Introduction

DNA replication is an essential process that requires precise regulation to duplicate the genome faithfully. Accurate and complete DNA replication relies on a balance between the rate of replication origin firing and the speed of replication fork progression [1–3]. Endogenous and exogenous sources of replication stress challenge these processes, and failure to respond to stalled replication forks leads to genome instability [4]. Post-translational modifications (PTMs) play a central role in responding to replication stress, with phosphorylation by ataxia telangiectasia and Rad3-related (ATR) kinase as one of the key steps. When the replisome encounters obstacles, single-stranded DNA (ssDNA) is exposed and coated by replication protein A (RPA). This activates ATR to initiate a phosphorylation cascade through DNA checkpoint kinase 1 (CHK1) to restrain the activities of cyclin-dependent kinases (CDKs). This cascade inhibits origin firing to halt replication and cell cycle progression, thus allowing appropriate repair to occur at stressed replication forks [5–7].

The additions of ubiquitin and small ubiquitin-like modifier (SUMO) are two indispensable PTMs during unperturbed DNA replication and the replication stress response. Both are conjugated to lysine residues on target proteins in a three-step cascade, requiring an E1 activating enzyme, an E2 conjugating enzyme and an E3 ligase [8]. Active replisomes are rich in SUMO but poor in ubiquitin PTMs, and this chemical environment is critical in sustaining fork progression [9]. Ubiquitination regulates mechanisms to mitigate replication stress, such as the activation of translesion DNA polymerases [10–12] or the stimulation of ATR kinase through RPA ubiquitination [13,14]. Ubiquitination is best characterized for its critical role in promoting the degradation of proteins and also the extraction of chromatin-bound proteins. Some targets include replication licensing factors that prevent re-replication [15,16] or replisome components that need to be removed upon replication termination or DNA damage [17,18]. Similarly, SUMO PTMs are rapidly stabilized upon genotoxic stress, leading to the activation of stress responses [19–21]. Protein SUMOylation modulates protein–protein or protein–DNA interactions, subcellular localizations, enzymatic activities or protein stabilities [22]. SUMOylation is critical in double-strand DNA break (DSB) repair [21,23,24] and protects stressed replication forks from excessive reversal or breakage [20,25]. Because they may share target lysines, ubiquitination and SUMOylation can be competitive in determining which repair pathway is activated [26,27]. However, these PTMs can be cooperative, thanks to the presence of SUMO-targeted E3 ubiquitin ligases (STUbLs) and SUMO deubiquitinases (SDUBs).

STUbLs are a unique class of E3 ligases that recognize SUMOylated proteins by their SUMO-interacting motifs (SIM) and catalyse the ubiquitination of substrate proteins through their really interesting new gene (RING) domain, leading to proteasomal degradation [28–30]. Mammalian cells only express two STUbLs, ring finger protein 4 (RNF4) and RNF111. RNF4 dimerizes through its RING domains upon binding to SUMOylated proteins [31–33], whereas RNF111 acts as a monomer [34,35]. STUbLs participate in diverse molecular processes and contribute to maintaining genome integrity [36]. For instance, *Saccharomyces cerevisiae* STUbL synthetic lethal with unknown [X] function (Slx) 5 and 8 (Slx5/Slx8) facilitates the relocalization of irreparable DSBs or stalled replication forks, which are heavily SUMOylated, to nuclear pores for repair or restart [36]. It remains unclear whether mammalian STUbLs are involved in similar processes. STUbLs also cooperate with cell cycle defective protein 48 (Cdc48, or p97), a molecular segregase that liberates proteins from higher-order complexes or chromatin [37,38]. Their cooperation ensures proper DNA interstrand cross-link (ICL) repair and limits illegitimate processing of replication-associated recombination intermediates [39,40]. Although several individual targets of human RNF4 or yeast Slx5/Slx8 have been identified, a comprehensive understanding of their function in DNA replication has not been systematically explored.

SDUBs deubiquitinate hybrid SUMO-ubiquitin chains to counteract the action of STUbLs. Two mammalian deubiquitinases, ubiquitin specific peptidase 7 (USP7) and USP11, have SDUB activity [9,41], and both are involved in multiple genome maintenance pathways [42,43]. USP7 is best characterized for its role as a molecular switch to selectively stabilize the mouse double minute 2 homologue (MDM2) E3 ubiquitin ligase or tumour suppressor p53 under normal conditions or upon DNA damage [44,45]. Therefore, USP7 plays a central role in determining the suppression or activation of p53-mediated apoptotic pathways under different circumstances [43]. Furthermore, the SDUB activity of USP7 is critical in maintaining the SUMO-rich, ubiquitin-poor environment surrounding active replisomes [9]. Inhibition of USP7 results in the accumulation of ubiquitin on SUMOylated proteins, displacing them from the replisome or causing p97-dependent degradation [9,46]. Although the enzymatic activities of STUbLs and SDUBs counteract each other, how these enzymes cooperate in certain biological processes (BP) remains elusive.

In this study, we used CRISPR–Cas9 genetic screens to reveal the genetic interaction (GI) profile of *RNF4* in non-transformed telomerase immortalized retinal pigment epithelial (hTERT RPE-1) cells. We found that *RNF4* mutants relied on *USP7* for survival, and cells lacking both RNF4 and USP7 exhibited reduced DNA synthesis and increased genomic instability. Moreover, *RNF4^−/–^/USP7^−/–^* mutants were hypersensitive to proteasome inhibition. Specifically, bortezomib, an FDA-approved therapeutic that inhibits 26S proteasome activity, caused significantly dysregulated DNA replication with increased replication fork asymmetry. Notably, bortezomib reduced RPA ubiquitination, which suppressed the ATR-mediated checkpoint response. *RNF4^−/–^/USP7^−/^*^−^ mutants were particularly vulnerable to bortezomib-mediated ATR suppression, leading to increased anaphase abnormalities and loss of viability. These findings reveal cooperation between RNF4 and USP7 in regulating the nuclear ubiquitin pool and genomic integrity.

## 2. Results

### 2.1. Genetic screens with a targeted CRISPR/Cas9 library reveal a genetic interaction profile for *RNF4*

**Figure 1.**
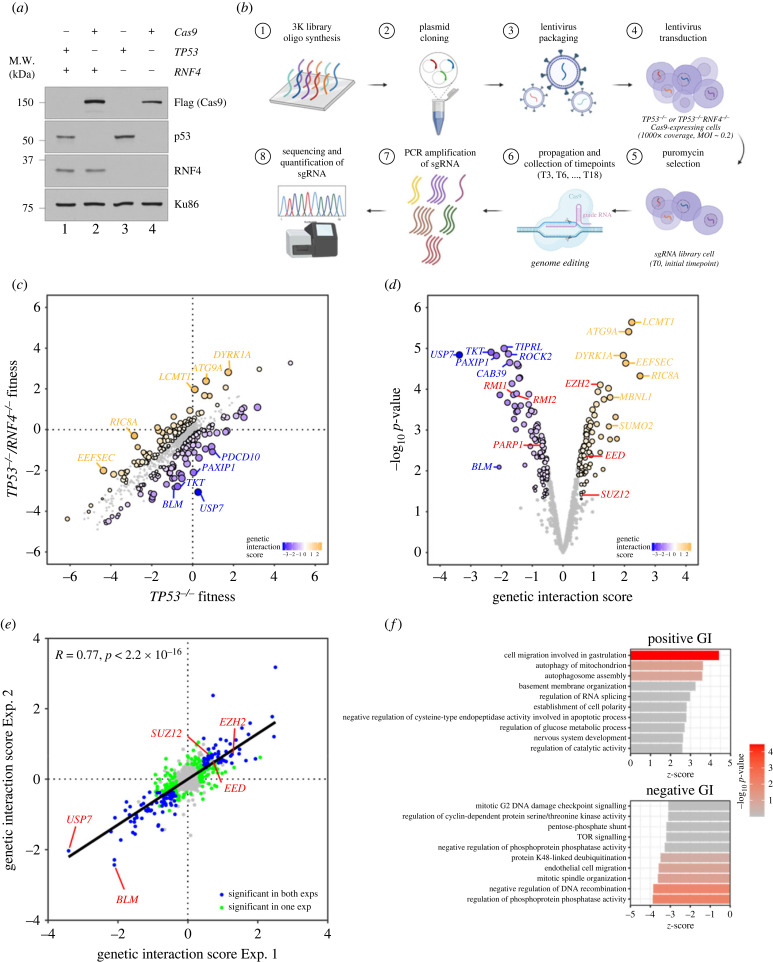
Genetic screens in *TP53*^−/–^ *RNF4*-proficient and *RNF4*-deficient cells. (*a*) Western blot analyses of whole cell extracts from RPE-1 WT, *RNF4^−/–^* and *TP53^−/–^* mutants with Cas9 expression. Lanes 2 and 4 are samples isolated from the cell lines used in the genetic screens. (*b*) Flow chart of the CRISPR–Cas9 genetic screen in RPE-1 *TP53^−/–^ RNF4*-proficient and RNF4-deficient cells using a custom, targeted DNA damage response sgRNA library. (*c*) Scatter plot showing the fitness of *RNF4*-proficient and -deficient cells between T0 and T18. The fitness is calculated as the log_2_ fold change of each gene between T0 and T18. Positive and negative genetic interactors are indicated in orange and blue, respectively. (*d*) Volcano plot showing the positive and negative genetic interactors of *RNF4* at T18. The genetic interaction scores are the differential fitness between *RNF4*-proficient and -deficient cells. Positive and negative genetic interactors are indicated in orange and blue, respectively. Other genes of interest are shown in red. (*e*) Scatter plot showing the genetic interaction scores in two independent biological experiments for each gene. The Pearson correlation coefficient (*R*) and *p*-value are indicated. (*f*) Gene ontology (GO) analysis of genetic interactors (GIs) of *RNF4* at T18. The top 10 positive (top) and negative (bottom) GO:BP terms and their *z*-scores are shown (see Material and methods). The significance (−log_10_ *p*-value) of each term is indicated by the colour scale.

To study the function of RNF4 in human cells, we generated *RNF4* KOs in RPE-1 cells using CRISPR–Cas9 targeting exon 3 of *RNF4* (electronic supplementary material, figure S1A,B). *RNF4^−/–^* cells were not hypersensitive to the replication inhibitors hydroxyurea (HU) or aphidicolin (APH) (electronic supplementary material, figure S1C,D). To understand whether other genes compensate for the loss of *RNF4*, we created double KOs with the only other human STUbL, *RNF111*, or the cell cycle regulator *TP53*. We generated *RNF111^−/–^*, *RNF4^−/–^/RNF111^−/^*, *TP53^−/–^* and *TP53^−/–^/RNF4^−/–^* cell lines (figure 1*a*). Although the loss of *RNF111* did not sensitize *RNF4^−/–^* to HU, knocking out *TP53* rendered *RNF4^−/–^* hypersensitive to HU and APH, suggesting that functional cell cycle checkpoints are required for *RNF4^−/–^* cells to respond to replication inhibition (electronic supplementary material, figure S1E,F).

To uncover other genes that are synthetically lethal with *RNF4^−/–^*, we performed genetic screens using a targeted CRISPR/Cas9 library in *RNF4*-proficient and -deficient RPE-1 cells. These screens were performed in a *TP53*-negative background, as it has been proposed that p53-mediated cell cycle arrest upon Cas9-induced DSB induction confounds the identification of essential genes in dropout genetic screens [47,48]. The CRISPR/Cas9 library contains sgRNAs targeting 1011 genes, with three sgRNAs per gene (a total of 3033 sgRNAs), including 350 DNA damage response (DDR) genes. Cas9 was constitutively expressed in *TP53^−/–^* and *TP53^−/–^/RNF4^−/–^* cells (figure 1*a*) [49]. Both cell lines had comparable Cas9 cutting efficiencies (electronic supplementary material, figure S1G), evaluated by transfecting chemically modified sgRNAs followed by tracking of indels by decomposition (TIDE) analysis [50]. Next, *TP53^−/–^* and *TP53^−/–^/RNF4^−/–^* cells were transduced with the CRISPR/Cas9 lentivirus library at a multiplicity of infection (MOI) of 0.2 and a 1000X representation of the sgRNA library. After puromycin selection, the library cells were collected (‘T0’ sample) and split into three independent replicates. Each replicate was collected and propagated every 3 days until day 18 (‘T18’ sample). Genomic DNA was extracted from each sample and the abundances of sgRNAs were quantified by next-generation sequencing (NGS) (figure 1*b*).

The NGS data were analysed using an adapted version of the Orthrus pipeline to score GIs [51] (see Material and methods). Briefly, changes in cell fitness were calculated on the sgRNA level as the difference in the normalized read counts between the start and the endpoint on a log_2_ scale. Guide-level GI scores were defined as the differences in cell fitness between *TP53^−/–^* and *TP53^−/–^/RNF4^−/–^* contexts. Gene-level GI scores were calculated as the average of the three guide-level GI scores for each gene, and each gene's GI score was tested for statistical significance (figure 1*c*,*d*) (see Material and methods). Positive or negative GI scores indicated that the loss of the gene either promoted or reduced cell growth in *TP53^−/–^/RNF4^−/–^* cells relative to *TP53^−/–^* cells (figure 1*d*). Screen quality was monitored by calculating the correlation between replicates and the recovery of expected essential gene dropout [49]. The Pearson correlation coefficients (PCCs) between replicates were high (greater than 0.9) throughout the dataset, and an area under the curve (AUC) of the receiver operating characteristic (ROC) curve above 0.85 suggested a robust identification of essential gene dropout (electronic supplementary material, figure S2). We determined that targeted genes with a false discovery rate (FDR) < 0.2 and an absolute GI score greater than 0.5 were significant candidates, including 113 positive and 135 negative genetic interactors (figure 1*d*; electronic supplementary material, table S1). Importantly, we performed an independent replicate screen and found that the gene-level GI scores across replicate experiments were highly correlated, with a PCC of 0.77 (figure 1*e*).

The strongest negative genetic interactor of *RNF4* was *USP7*, a SDUBs. Other negative interactors included targets involved in ubiquitin metabolism, such as deubiquitinase *OTUB1*, *USP9X* and *USP15*, and ubiquitin E1 (*UBA5*), E2 (*UBE2H* and *UBE2T*), and E3 enzymes (*UBA5*, *UBE2H*, *UBE2T*, *RNF10*, *RNF111*, *RNF138*, *CUL5*, *RFWD2* and *SYVN1*). Additionally, we identified several genes encoding components of the BLM-TOP3A-RMI1-RMI2 (BTRR) complex as negative genetic interactors, consistent with the fact that yeast *Slx5/Slx8* is synthetically lethal with *Sgs1*, the yeast counterpart of *BLM* [52]. The telomere maintenance factors *POT1* and *TEN1* were also synthetically lethal with *RNF4*, consistent with the recent CRISPR/Cas9 screens where *RNF4* was identified as synthetically lethal with mutant *POT1* in human cells [53]. Mitotic checkpoint genes (*TAOK1*, *STAG1*, *STAG2* and *MAD2L2*), cell cycle regulators (*CCNA1*, *CCNC*, *CDK4* and *CDC25A*) and DNA replication factors (*MCM3* and *CDC45*) were among the negative genetic interactors of *RNF4* (electronic supplementary material, table S1).

One of the top positive genetic interactors was *SUMO2*, depletion of which improved cell growth in the *TP53^−/–^/RNF4^−/–^* cells, supporting the notion that the accumulation of SUMO conjugates reduces cell viability [54]. We also identified several genes encoding proteins participating in nuclear pore transport, including *RANBP1*, *LMNA* and *TNPO1*. Studies in yeast suggest that Slx5/Slx8 facilitates the relocation of damaged DNA to the nuclear periphery for repair [36]. Our finding that genes associated with nuclear pore transport are positive genetic interactors of *RNF4* further support that RNF4 may participate in a similar pathway in human cells. Additionally, we identified several genes encoding proteins involved in chromatin remodelling and histone modification, including the ATRX/DAXX complex (*ATRX* and *DAXX)*, the polycomb repression complex (*EED*, *EZH2* and *SUZ12*), histone methyltransferases (*SUV39H1* and *KMT2A*), histone demethylase (*KDM5C*) and histone acetylase and deacetylase (*CREBBP* and *HDAC3*) (electronic supplementary material, table S1).

To systematically analyse the GIs of *RNF4*, we summarized GIs at the level of gene ontology (GO) terms. The genes targeted by our CRISPR/Cas9 library span 1186 GO-BP terms, in which we only included terms represented by at least three library genes. We computed a *z*-score per GO:BP term to measure the direction and strength of interactions of the member genes, and FDR was calculated, using Benjamini–Hochberg (BH) multiple testing correction. Cell migration involved in gastrulation (*AMOT*, *LRP5* and *RIC8A*) and autophagy-related pathways (*ATG9A*, *MARK2*, *RB1CC1*, *USP30*, *RB1CC1* and *VMP1*) were enriched as positive genetic networks of *RNF4*, suggesting that RNF4 might play a role in regulating these processes (figure 1*f*). By contrast, regulation of phosphoprotein phosphatase activity (*PPP6R3*, *PTPA*, *TIPRL* and *TSC1*), suppression of DNA recombination (*BLM*, *H1-2*, *MSH2*, *MSH3* and *MSH6*), mitotic spindle organization (*KIF4A*, *PTPA*, *SBDS* and *TNKS*), endothelial cell migration (*PAXIP1*, *PIK3CA* and *PTEN*) and protein K48-linked deubiquitination (*BAP1*, *OTUB1*, *OTUD5*, *USP15*, *USP33*, *USP7* and *USP9X*) were enriched in negative genetic networks (figure 1*f*) of *RNF4*. Notably, K48- or K63-linked deubiquitination pathways were enriched throughout the time course of the screens (T6, T12 and T18), suggesting that cells need alternative pathways to metabolize polyubiquitinated proteins when RNF4 is absent (electronic supplementary material, table S2).

In summary, using a DDR-focused targeted CRISPR/Cas9 library, we uncovered robust genetic networks representing alternative pathways that *RNF4* mutants rely on for processing polyubiquitinated proteins.

### 2.2. Depletion of *USP7* in *RNF4* knockouts reduces cell growth and increases genome instability

**Figure 2.**
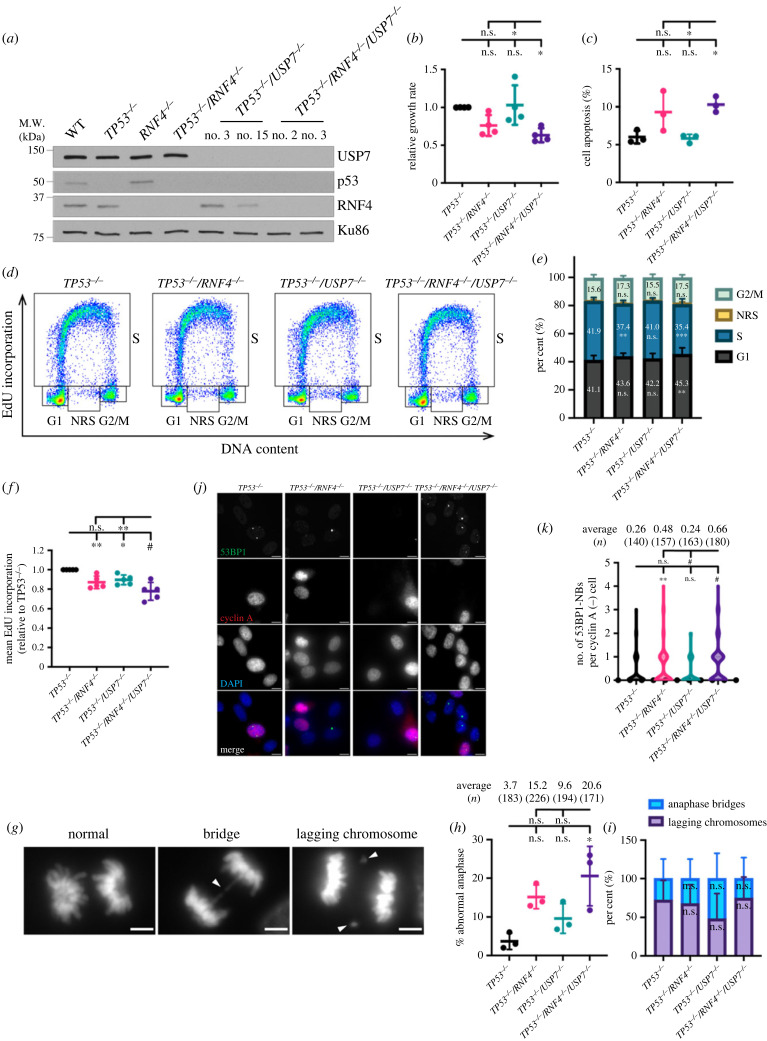
*USP7* is synthetically sick with *RNF4* deficiency. (*a*) Western blot of whole cell extracts from RPE-1 WT, *RNF4^−/–^*, *TP53^−/–^*, *TP53^−/–^/RNF4^−/–^*, *TP53^−/–^/USP7^−/–^* and TKO cells. (*b*) Proliferation rate in *RNF4* and *USP7* mutants normalized to *TP53^−/–^* cells. (*c*) Cell apoptosis measured by Annexin V-PI staining followed by FACS analysis. (*d*) Representative cell cycle distributions based on DNA content (DAPI) and DNA synthesis (EdU incorporation). Cell cycle phases (G1, S, G2/M and NRS) are indicated. (*e*) Cell cycle distribution of each cell line. Cumulative bars representing the average percentage of G1-, S-, G2/M- or NRS-phase populations from three experiments are shown. Error bars indicate standard deviations (SDs). Significance was measured using a two-way ANOVA with the Geisser–Greenhouse correction and Šidák's multiple comparisons test, n.s.: not significant, **p ≤ 0.01, ^#^*p* ≤ 0.0001. (*f*) DNA synthesis in S-phase cells measured by EdU/DAPI staining and normalized to *TP53^−/–^* cells. (*g*) Representative images of normal and abnormal anaphases. White arrows indicate the lagging chromosome or the anaphase bridge. The scale bar is 5 µm. (*h*) Anaphase abnormalities scored by examining anaphase cells with DAPI staining. Bars represent the average of three biological experiments and the total numbers of anaphases scored are shown. Significance was measured using an ordinary one-way ANOVA with Tukey's multiple comparisons test, n.s.: not significant, *p ≤ 0.05. (*i*) The breakdown of abnormal anaphases in each cell line. Cumulative bars representing the average percentage of anaphase bridges or lagging chromosome from three experiments are shown. Error bars indicate SD. Significance was measured using an ordinary two-way ANOVA with Šidák's multiple comparisons test, n.s.: not significant. (*j*) Representative images of 53BP1-NB immunofluorescent staining. The scale bar is 10 µm. (*k*) Violin plot showing the number of 53BP1-NBs in G1 (cyclinA-negative) cells. The total numbers of G1 cells scored from two independent experiments are shown. Significance was measured using a Kruskal–Wallis with Dunn's multiple comparisons test, n.s.: not significant, **p ≤ 0.01, ^#^≤ 0.0001. (*b*,*c*,*f*) Each data point represents the average value from independent plates in an experiment. Bars and error bars indicate the mean and SD across multiple biological experiments. Significance was measured using an ordinary one-way ANOVA with Tukey's multiple comparisons test; n.s., not significant; *p ≤ 0.05; **p ≤ 0.01; ^#^*p* ≤ 0.0001.

To validate the negative GI between *RNF4* and *USP7*, we generated *USP7* KOs in both *TP53^−/–^* and *TP53^−/–^/RNF4^−/–^* backgrounds (figure 2*a*). Whereas knocking out *USP7* in the *TP53^−/–^* cells (hereafter *TP53^−/–^/USP7^−/–^*) did not affect cell growth, loss of *USP7* in the *TP53^−/–^/RNF4^−/–^* cells (hereafter *TP53^−/–^/RNF4^−/–^/USP7^−/–^* triple KO, TKO) significantly decreased cell proliferation (figure 2*b*), validating the genetic screen data. The slower proliferation rate in the TKO cells was partly due to increased apoptosis (figure 2*c*). To investigate other causes of the growth defect, we performed quantitative single cell flow cytometry to assess cell cycle distribution and S-phase DNA synthesis. Cell cycle distribution was determined by staining with 4′,6-diamidino-2-phenylindole (DAPI) for DNA content in combination with a pulse of 5-ethynyl-2′-deoxyuridine (EdU) to label S-phase cells (figure 2*d*). In addition to G1-, S- and G2-phase cells, non-replicating S (NRS)-phase cells were defined as cells whose DNA content was between 2N and 4N but did not incorporate detectable levels of EdU. We observed a small but significant increase (45.3% versus 41.1%) in G1- and a decrease (35.4% versus 41.9%) in S-phase cells in the TKO compared to *TP53^−/–^* cells (figure 2*e*), indicating delayed progression through the G1/S transition in TKO cells. Furthermore, the mean EdU intensity in S-phase cells was significantly lower (78.4%) in the TKO than in *TP53^−/–^* cells, suggesting that TKO cells have DNA synthesis defects (figure 2*f*). Certain genomic loci are intrinsically hard to replicate, such as common fragile sites (CFSs), and are particularly susceptible to under-replication and forming persistent late replicating intermediates (LRIs) if cells have defective DNA synthesis [55]. LRIs that are unresolved before cells enter anaphase can result in anaphase bridges [56]. Incomplete replication can also lead to lagging of whole chromosomes or acentric chromosome fragments [57,58]. To evaluate whether the DNA synthesis defects resulted in mitotic errors, we analysed the frequency of anaphase abnormalities including DAPI-positive bridges and lagging chromosomes (figure 2*g–i*). The TKO cells had a significantly higher (20.6% versus 3.7%) frequency of anaphase abnormalities than *TP53^−/–^* cells (figure 2*h*). The proportions of each type of abnormal anaphases did not significantly differ in any genotype (figure 2*i*). In addition to causing anaphase defects, under-replicated regions can be transmitted to daughter cells when sequestered in G1-phase p53 binding protein 1 (53BP1) nuclear bodies (NBs), which are resolved in the following S-phase [59,60]. Indeed, G1-phase TKO cells had a significantly higher average number of 53BP1-NBs than *TP53^−/–^* cells (0.66 versus 0.26; figure 2*j–k*). Notably, *TP53^−/–^/RNF4^−/–^* mutants exhibited moderate reduction in DNA synthesis and increase in 53BP1-NBs compared to *TP53^−/–^* cells, but were further exacerbated in the TKO cells. Together, these data suggest that USP7 is required for *TP53^−/–^/RNF4^−/–^* cells to maintain genomic stability and survival.

### 2.3. *TP53^−/–^/RNF4^−/–^/USP7^−/–^* triple knockouts are hypersensitive to proteasome inhibitor

**Figure 3.**
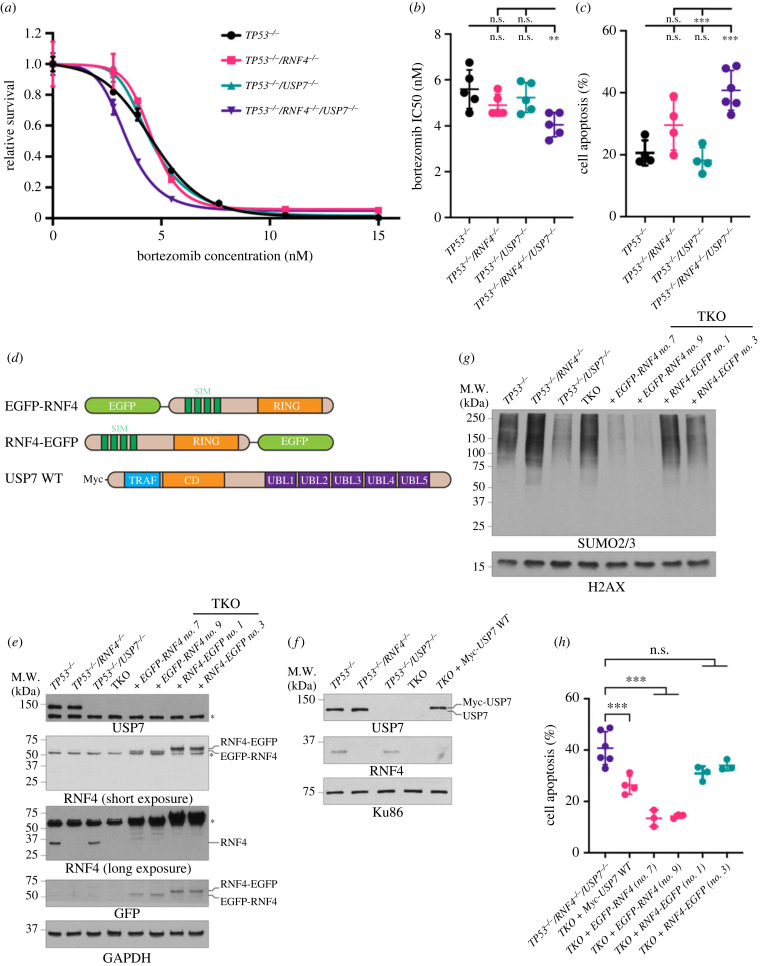
Bortezomib causes elevated cell apoptosis in TKO cells. (*a*) Representative dose–response curve to bortezomib measured by CellTiter-Glo cell viability assay. Cell survival is normalized to the untreated control. The mean survival with SD of each concentration is shown. The asymmetrical (five-parameter) logistic dose–response model is used to fit the curve. (*b*) Bortezomib IC50 values. Each data point is an IC50 value from an experiment. Bars and error bars indicate the mean with SD. (*c*) Cell apoptosis after 48 h of bortezomib measured by Annexin V-PI staining. Each data point is a biological replicate and the mean with SD is shown. (*d*) Cartoon schematics of constructs used to complement TKO cells. EGFP, enhanced green fluorescent protein; SIM, SUMO-interacting motif; RING, really interesting new gene; TRAF, tumour necrosis factor receptor-associated factor; CD, catalytic domain; UBL, ubiquitin-like. (*e*) Western blot analyses of whole cell extracts of TKO cells expressing either EGFP-RNF4 or RNF4-EGFP. (*f*) Western blot analyses of whole cell extracts of TKO cells expressing WT USP7. (*g*) Western blot analyses of SUMOylated chromatin-bound proteins of TKO cells expressing either EGFP-RNF4 or RNF4-EGFP. Cells were treated with 10 µM of MG132 and 1 mM of HU for 4 h before collecting. (*h*) Cell apoptosis after 48 h of bortezomib measured by Annexin V-PI staining in TKO and complemented cells. (*b*,*c*,*h*) Each data point represents the average value from independent plates in an experiment. Bars and error bars indicate the mean and SD across multiple biological experiments. Significance was measured using an ordinary one-way ANOVA with Tukey's multiple comparisons test; n.s., not significant; **p ≤ 0.01; ***p ≤ 0.001.

Since both RNF4 and USP7 are critical for regulating ubiquitin metabolism, we hypothesized that TKO cells may be vulnerable to proteasome inhibition. While losing either *RNF4* or *USP7* insignificantly increased sensitivity to bortezomib, an FDA-approved therapeutic for multiple myeloma and mantel cell lymphoma [61], losing both genes rendered cells significantly more sensitive (figure 3*a–c*). TKO cells had a significantly lower IC50 value (4.05 versus 5.59 nM) (figure 3*a*,*b*) and a significant increase in apoptosis (41.5% versus 20.6%) compared to *TP53^−/–^* cells (figure 3*c*). Importantly, TKO cells were hypersensitive to other proteasome inhibitors, including MG132 and two clinical inhibitors, ixazomib and carfilzomib [62] (electronic supplementary material, figure S3*a–c*). By contrast, TKO cells were not hypersensitive to CB-5083, a p97 segregase inhibitor [63] (electronic supplementary material, figure S3D), indicating that p97-dependent protein turnover remains unaffected in the mutants. Interestingly, the increased apoptosis induced by bortezomib was not due to elevated proteotoxic stress in TKO cells, as all four cell lines exhibited comparable levels of unfolded proteins upon bortezomib treatment (electronic supplementary material, figure S3E). These data suggest that TKO cells are susceptible to proteasome inhibition rather than the inability to remove chromatin-bound proteins or to metabolize unfolded proteins.

To verify that sensitivity to bortezomib was indeed due to the loss of *RNF4* or *USP7*, we performed complementation experiments in TKO cells. We reasoned that a C-terminal enhanced green fluorescent protein (EGFP) tag on RNF4 (RNF4-EGFP) would interfere with the dimerization of the RING domain [31,32] and be non-functional, whereas N-terminally tagged RNF4 (EGFP-RNF4) would be functional (figure 3*d*). The expression levels of these transgenes were examined by western blot (figure 3*e*,*f*). As expected, TKO cells complemented with EGFP-RNF4, but not RNF4-EGFP, had significantly reduced levels of SUMOylated proteins on chromatin upon replication stress (figure 3*g*), supporting that EGFP-RNF4 and RNF4-EGFP are active and inactive forms of RNF4, respectively. Interestingly, while complementing with USP7 modestly restored viability in TKO cells, complementing with EGFP-RNF4 significantly improved the viability of TKO cells treated with bortezomib. Conversely, RNF4-EGFP complementation did not rescue viability of TKO cells (figure 3*h*). These data suggest that functional RNF4 and USP7 are critical for TKO cells to suppress apoptosis induced by bortezomib and that RNF4 plays a dominant role in modulating cell sensitivity.

### 2.4. Bortezomib significantly reduces DNA synthesis in the *TP53^−/–^/RNF4^−/–^/USP7^−/–^* mutants

**Figure 4.**
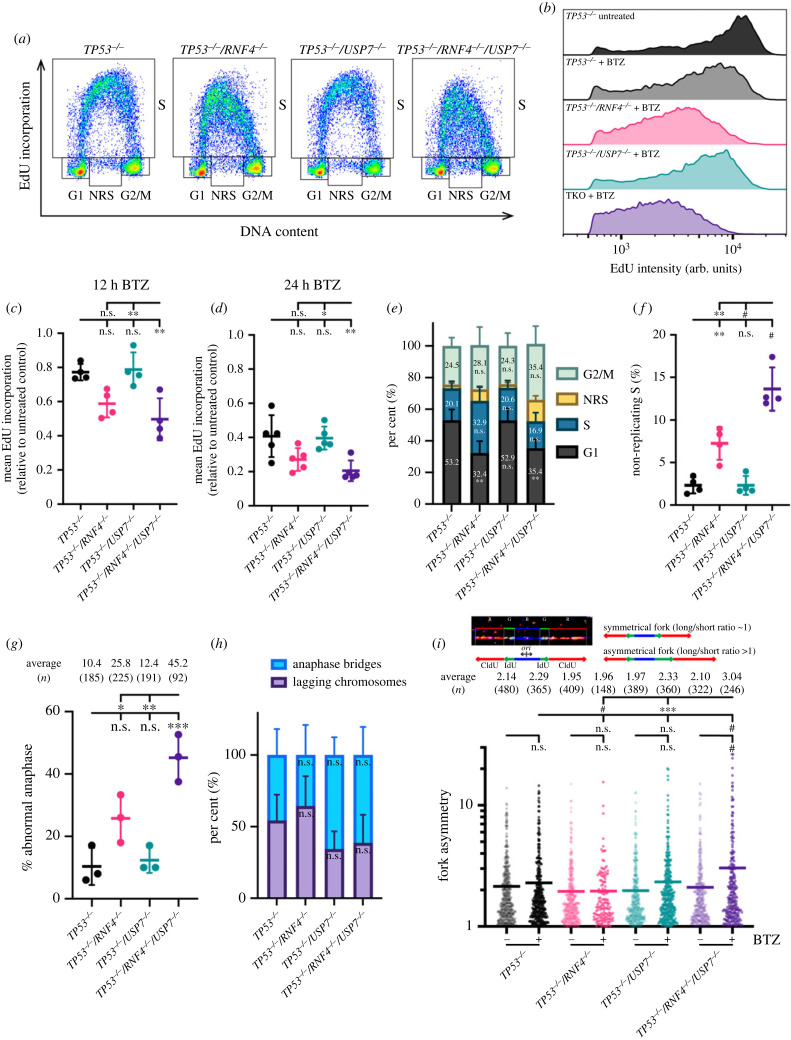
DNA synthesis is significantly reduced in TKO treated with bortezomib. (*a*) Representative cell cycle distributions based on DNA content (DAPI) and DNA synthesis (EdU incorporation) after 12 h of BTZ treatment. Cell cycle phases (G1, S, G2/M and NRS) are indicated. (*b*) Representative histograms of DNA synthesis in S-phase cells. Cells were incubated with or without BTZ for 12 h. (*c*,*d*) DNA synthesis in S-phase cells measured by EdU/DAPI staining and normalized to the untreated control. Cells were treated with BTZ for 12 h (*c*) or 24 h (*d*). (*e*) Cell cycle distribution of each cell line treated with BTZ for 24 h. Cumulative bars representing the average percentage of G1-, S-, G2/M- or NRS-phase populations are shown. Error bars indicate SD. Significance was measured using a two-way ANOVA with the Geisser–Greenhouse correction and Šidák's multiple comparisons test, n.s.: not significant, **p ≤ 0.01. (*f*) Percentage of NRS-phase cells in each cell line treated with BTZ for 24 h. (*g*) Anaphase abnormalities scored by DAPI staining following treatment with BTZ for 24 h. Representative images are shown in figure 2*g*. Bars represent the average of three biological experiments and the total numbers of anaphases scored are shown. (*c*,*d*,*f*,*g*) Ordinary one-way ANOVA with Tukey's multiple comparisons test, n.s.: not significant, *p ≤ 0.05, **p ≤ 0.01, ***p ≤ 0.001, ^#^*p* ≤ 0.0001. (*h*) The breakdown of abnormal anaphases in each cell line. Cumulative bars representing the average percentage of anaphase bridges or lagging chromosome from three experiments are shown. Error bars indicate SD. Significance was measured using an ordinary two-way ANOVA with Šidák's multiple comparisons test, n.s.: not significant. (*i*) Fork asymmetry analysed by DNA combing. Fork asymmetry was calculated as the ratio of the long track over the short track of sister forks flanking each origin (ori). Top, representative sister forks; bottom, quantification of fork asymmetry in cells incubated with or without BTZ for 12 h. Average fork asymmetry and the number of sister forks quantified (*n*) are listed. Significance was measured using a one-way Kruskal–Wallis test with Dunn's multiple comparisons tests; n.s., not significant; ***p ≤ 0.001; ^#^*p* ≤ 0.0001.

It has been shown that bortezomib decreases the levels of homologous recombination (HR) proteins and depletes the nuclear ubiquitin pool. Therefore, bortezomib interferes with DSB repair, and sensitizes multiple myeloid cells to poly(ADP-ribose) polymerase (PARP) inhibitors [64]. However, with 12 h of bortezomib treatment, we did not observe a reduction in Fanconi anaemia (FA) complementation group D2 (FANCD2) or RAD51 (electronic supplementary material, figure S4A), two factors that were depleted in multiple myeloid cells upon similar treatment [64]. Additionally, the *γ*H2AX levels were comparable after 48 h of bortezomib treatment in either *RNF4* or *USP7* mutants (electronic supplementary material, figure S4B). Thus, it is unlikely that the sensitivity to bortezomib in TKO cells was due to defects in repairing endogenous DSBs. To uncover the cause of increased sensitivity to bortezomib, we assessed bulk DNA synthesis upon bortezomib treatment (figure 4*a*). Surprisingly, although 12 h of bortezomib treatment caused a relatively mild reduction (by 23%) in DNA synthesis in *TP53^−/–^* cells, the synthesis rate was markedly reduced (by 50%) in TKO cells (figure 4*b*,*c*). Furthermore, the reduction in DNA synthesis worsened over time, with a rate reduced by 60% and 80% compared to untreated control in *TP53^−/–^* and TKO cells after 24 h of bortezomib treatment, respectively (figure 4*d*). While the reduction in DNA synthesis in TKO cells was significantly impaired when compared to *TP53^−/–^/USP7^−/–^* mutants, it was not significantly different from *TP53^−/–^/RNF4^−/–^* mutants, suggesting that RNF4 plays a major role in sustaining DNA synthesis and USP7 functions in a backup pathway (figure 4*c*,*d*). The reduction in DNA synthesis led to a slightly extended S-phase in *RNF4* mutants, although this was not statistically significant (figure 4*e*). Notably, after 24 h of bortezomib treatment, TKO cells had an approximately 6-fold increase in NRS-phase cells compared to *TP53^−/–^* cells (13.6% versus 2.3%), and was also significantly higher than either the *TP53^−/–^/USP7^−/–^* or *TP53^−/–^/RNF4^−/–^* double mutants (figure 4*f*). If cells with incomplete DNA synthesis still progressed into mitosis, then the frequency of anaphase abnormalities was predicted to significantly increase. In fact, TKO cells had a significant increase in anaphase abnormalities in comparison to all other mutants, including a 4-fold elevation when compared to *TP53^−/–^* cells (45.2% versus 10.4%) (figure 4*g*). Notably, the proportion of each type of abnormal anaphases were comparable among all genotypes (figure 4*h*).

To further dissect the DNA synthesis defects caused by bortezomib on a single molecule level, we performed DNA combing. Cells were incubated with or without bortezomib for 12 h and then subjected to two sequential nucleoside analogue pulses in the presence or absence of the drug (electronic supplementary material, figure S4C). Replication fork progression was not significantly altered in *RNF4* or *USP7* mutants upon bortezomib treatment (electronic supplementary material, figure S4D). Although TKO cells had distinctly longer inter-origin distances (IODs) than *TP53^−/–^* cells under unperturbed conditions (103 versus 64.5 kb), bortezomib treatment did not further lengthen IODs in TKO cells (electronic supplementary material, figure S4E). Interestingly, upon bortezomib treatment, only TKO cells exhibited a significant rise in replication fork asymmetry (figure 4*i*), suggesting that replication forks are prone to stalling. The fact that fork asymmetry was significantly higher in the TKOs than all other mutants suggested that RNF4 and USP7 might be involved in parallel pathways to prevent replication fork stalling. Together, these data indicate that DNA synthesis in TKO cells is severely dysregulated when the proteasome is inhibited, leading to dramatically altered replication patterns.

### 2.5. Bortezomib suppresses ATR-mediated checkpoint response

**Figure 5.**
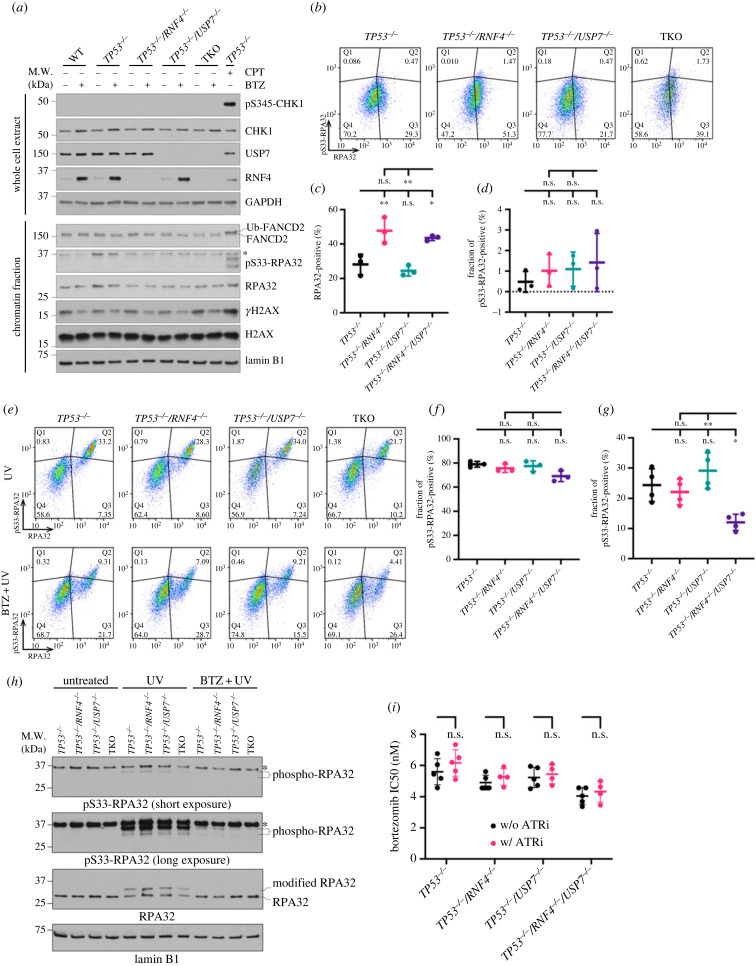
TKO cells are susceptible to bortezomib-mediated ATR suppression. (*a*) Western blot analyses of whole cell extracts and chromatin fractions of cells incubated without or with BTZ for 12 h. *TP53^−/–^* cells treated with 1 µM of camptothecin (CPT) for 12 h was used as a control for ATR activation. Asterisk indicates a non-specific band. (*b*) Representative analytic flow cytometry plots for chromatin-bound RPA32 and pS33-RPA32. Cells were incubated in BTZ for 12 h. (*c*) Quantification of RPA32-positive cells after 12 h of bortezomib treatment based on analytic flow cytometry. (*d*) Quantification of the fraction of pS33-RPA32-positive cells in total RPA32-positive cells as in (*c*). The fraction of pS33-RPA32-positive cells in total RPA32-positive cells was calculated as Q2/(Q2 + Q3) shown in (*b*). (*c*,*d*) Each data point represents the average value from independent plates in an experiment. Bars and error bars indicate the mean and SD across multiple biological experiments. Significance was measured using an ordinary one-way ANOVA with Tukey's multiple comparisons test, n.s.: not significant, *p ≤ 0.05, **p ≤ 0.01. (*e*) Representative analytic flow cytometry plots for chromatin-bound RPA32 and pS33-RPA32. Cells were grown with or without BTZ for 12 h prior to exposure to 100 J m^−2^ of UV and collected after 2 h. (*f*,*g*) Quantification of the fraction of pS33-RPA32-positive cells in total RPA32-positive cells with UV alone (*f*) or bortezomib plus UV (*g*). Each data point represents the average value from independent plates in an experiment. Bars and error bars indicate the mean and SD across multiple biological experiments. Significance was measured using an ordinary one-way ANOVA with Tukey's multiple comparisons test; n.s., not significant; *p ≤ 0.05; **p ≤ 0.01. (*h*) Western blot analyses of chromatin fractions of cells under untreated, UV alone or bortezomib plus UV treatment. (*i*) Bortezomib IC50 values in the presence or absence of 200 nM of ATR inhibitor. Each data point is an IC50 value from an experiment. Bars and error bars indicate the mean with SD. Significance was measured by ordinary two-way ANOVA with Šidák's multiple comparisons test; n.s., not significant.

Despite the reduced DNA synthesis upon bortezomib treatment, cells did not activate ATR, as we did not detect phospho-CHK1 at S345 (pS345-CHK1), phospho-RPA32 at S33 (pS33-RPA32), or FANCD2 ubiquitination (figure 5*a*; electronic supplementary material, figure S4B). Using quantitative flow cytometry analysing chromatin-bound proteins, we observed that TKO cells had a more than 1.5-fold increase in RPA32-positive cells compared to *TP53^−/–^* mutants upon proteasome inhibition. Only a small fraction (less than 5%) of RPA32 was phosphorylated (figure 5*b–d*). It has been shown that RFWD2-mediated RPA ubiquitination is a prerequisite for ATR-mediated RPA phosphorylation and that proteasome inhibition suppresses RPA ubiquitination [14]. Therefore, we hypothesized that TKO cells are particularly susceptible to bortezomib-mediated suppression of RPA phosphorylation. To test this hypothesis, we incubated cells in the absence or presence of bortezomib for 12 h prior to UV exposure and analysed the fraction of pS33-RPA32 after 2 h (figure 5*e*). Upon UV exposure, most (greater than 70%) of the chromatin-bound RPA32 was phosphorylated (figure 5*f*). However, with bortezomib pretreatment, the fraction of pS33-RPA32 was significantly reduced, with TKO cells exhibiting the lowest fraction (12.6%) among the four cell lines (figure 5*g*). Using western blot, we observed a similar reduction in RPA32 phosphorylation when cells were treated with bortezomib prior to UV exposure (figure 5*h*). Lastly, when we combined bortezomib with ATRN-119, an ATR inhibitor, we did not find any additive or synergistic effect, supporting that proteasome and ATR activities are epistatic (figure 5*i*). Notably, the impairment of ATR signalling upon proteasome inhibition was independent of the source of replication stress and *TP53* status (electronic supplementary material, figure S5). Together, these data suggest that bortezomib restrains ATR activation and that TKO cells are highly vulnerable to bortezomib-mediated ATR suppression.

## 3. Discussion

**Figure 6.**
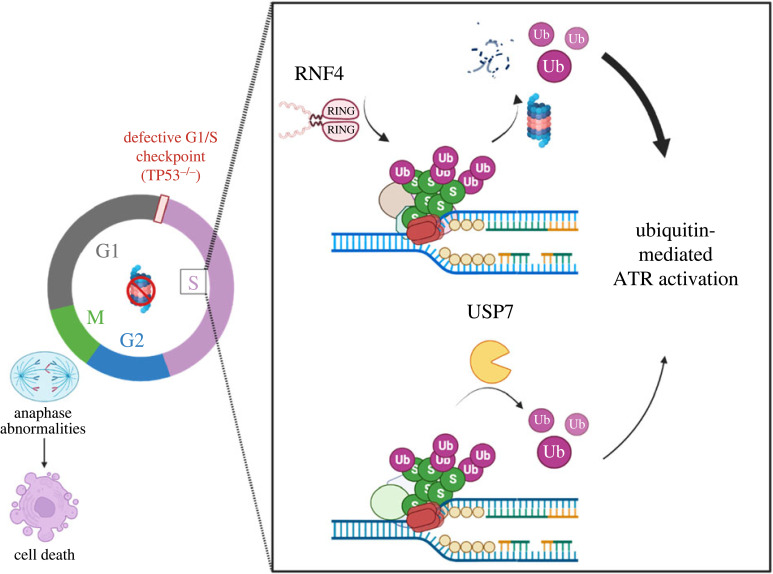
RNF4 and USP7 cooperate in ubiquitin-regulated steps of DNA replication. *TP53^−/–^* cells have defective G1/S checkpoint, leading to unrestrained G1/S transition even in the presence of DNA damage. With a functional intra-S checkpoint response, cells are capable of pausing and repairing the lesions to prevent premature mitotic entry if cells accumulate too much damage. RNF4, a SUMO-targeted E3 ubiquitin ligase, facilitates the turnover of SUMOylated and ubiquitinated proteins accumulated upon replication stress. USP7, on the other hand, maintains the SUMO and ubiquitin balance at the replication forks by deubiquitinating substrate proteins. These two seemingly opposite actions, however, both sustain the nuclear ubiquitin pool and are particularly critical when proteasome activity is inhibited, where RNF4 and USP7 play a major and a minor role, respectively. Depletion of the nuclear ubiquitin pool suppresses the ATR-mediated checkpoint response upon proteasome inhibition. TKO cells exhibit significantly increased fork asymmetry and reduced DNA synthesis when the proteasome is inhibited. A compromised ATR-mediated checkpoint upon proteasome inhibition permits premature mitotic entry in TKO cells, leading to high levels of anaphase abnormalities and presumably programmed cell death.

Using a targeted CRISPR/Cas9 library, we have revealed a DDR-focused GI profile for *RNF4*. Among the negative GIs, K48-linked deubiquitination was of particular interest, as it suggested that *RNF4* mutants rely on alternative pathways for metabolizing polyubiquitinated proteins. *USP7*, a SDUBs, was the strongest negative genetic interactor of *RNF4*. We verified this GI by generating *TP53^−/–^/RNF4^−/–^/USP7^−/–^* TKO cells, which exhibited reduced DNA synthesis and increased 53BP1-NBs (figure 2). Furthermore, TKO cells were hypersensitive to proteasome inhibitors (figure 3), which deplete the nuclear ubiquitin pool [64]. Upon proteasome inhibition, the DNA synthesis rate in TKO cells was severely reduced with nearly 45% of S-phase cells completely stopping replication. Consequently, nearly half (approx. 45%) of TKO cells treated with proteasome inhibitor exhibited anaphase abnormalities (figure 4). Lastly, we showed that ATR signalling was significantly compromised upon proteasome inhibition and that TKO cells were the most vulnerable to bortezomib-mediated ATR suppression (figure 5). We therefore propose a model in which RNF4 and USP7 work in parallel to replenish the nuclear ubiquitin pool, a prerequisite of functional ubiquitin-mediated checkpoint response, especially when the proteasome is inhibited (figure 6). Failure to activate the ATR-mediated checkpoint response results in premature mitotic entry when DNA replication is incomplete, and cells with high levels of anaphase abnormalities presumably undergo programmed cell death if chromosome damage persists.

It is intriguing that *RNF4* and *USP7* exhibited a strong negative GI, as these two proteins do not enzymatically complement each other. In fact, USP7 has been shown to directly deubiquitinate SUMO-ubiquitin chains catalysed by RNF4 *in vitro*, and thus it has been considered a SDUBs [9]. Furthermore, RNF4 and USP7 share common targets including topoisomerase 1 (TOP1) [65–67] and mediator of DNA damage checkpoint protein 1 (MDC1) [68,69], and their catalytic activities antagonize each other. However, it is not unprecedented that RNF4 and UPS7 can work in parallel and fulfil similar functions. For instance, USP7 and RNF4 both functionally contribute to the resolution of DNA–protein cross-links (DPCs). USP7 stabilizes SprT-like N-terminal domain protein (Spartan, SPRTN) [70], the key protease in resolving DPCs [71,72], whereas RNF4 clears SUMOylated DPCs through the proteasome [67]. In our study, we found that the simultaneous loss of *RNF4* and *USP7* significantly reduced DNA synthesis rate and increased genomic instability (figure 2). This negative GI between *RNF4* and *USP7* was p97-independent (electronic supplementary material, figure S3D). RNF4 and USP7 work in parallel to replenish the nuclear ubiquitin pool. RNF4 promotes the turnover of SUMOylated and ubiquitinated proteins, and USP7 facilitates protein deubiquitination. Therefore, our findings reveal another possible mechanism in which RNF4 and USP7 collaboratively maintain genome stability by controlling the functional ubiquitin pool, and their activities are critical when proteasome activity is inhibited.

USP7 has emerged as a key regulator of genome stability involved in multiple ubiquitination pathways that maintain appropriate cell cycle regulation, DNA replication and DSB repair [43]. USP7 is best characterized for its role in modulating MDM2 and p53 levels under both normal and stress conditions such as DNA damage [44,45]. Under stress conditions, USP7 preferentially deubiquitinates and stabilizes p53, allowing for the upregulation of downstream p53-targets such as CDK inhibitor 1 (p21^Cip1^) and the induction of cell cycle arrest or apoptosis [43]. When USP7 is inhibited in *TP53*-positive cells, the SUMO-rich environment at the replication fork is altered, resulting in slower replication fork progression and reduced origin firing [9]. Surprisingly, we did not observe any changes in the rate of DNA synthesis or replication fork speed in *TP53*-negative *USP7* mutants (figure 2*f*; electronic supplementary material, figure S4D). It is likely that the effect of inhibition of USP7 on replication fork progression that the previous study showed is partly through the stabilization of p53 and the activation of p21, a suppressor of replication fork speed [73]. Conversely, we found that both *TP53^−/–^/USP7^−/–^* and TKO cells had increased IODs (electronic supplementary material, figure S4E) indicative of reduced origin firing, suggesting that this phenotype is indeed induced by the loss of *USP7* and is p53-independent. In *TP53^−/–^/USP7^−/–^* mutants, when RNF4 is still present, DNA synthesis occurred normally without accruing under-replicated regions leading to subsequent anaphase abnormalities or 53BP1-NBs in the next cell cycle. By contrast, when *RNF4* was also lost, DNA synthesis was significantly reduced and caused the accumulation of under-replicated DNA (figure 2*d–k*). Taken together, these data suggest that RNF4 is responsible for sustaining DNA replication and repairing damage that occurs in *USP7* mutants.

Bortezomib depletes the nuclear pool of ubiquitin and impairs K63-linked polyubiquitination of H2AX, a requirement for the recruitment of BRCA1 and RAD51 to DSBs [74], leading to the accumulation of unrepaired breaks [64]. Neri *et al*. [64] also showed the transcripts of several HR genes, including *RAD51* and *FANCD2*, were downregulated upon bortezomib treatment. Both effects contributed to the inability to repair DSBs. Multiple reports have shown that bortezomib stabilizes and activates p53 signalling [75,76], which leads to E2F-dependent downregulation of FA genes, including canonical HR factors [77]. In our study, we did not observe reduction of RAD51 or FANCD2 protein levels (electronic supplementary material, figure S4A), which may be attributed to the absence of *TP53* in these cells. Furthermore, we did not observe differences in persistent *γ*H2AX among any of the *RNF4* or *USP7* mutants (electronic supplementary material, figure S4B), suggesting the hypersensitivity to bortezomib was not due to the inability to repair DSBs. Proteasome inhibition also results in the accumulation of unfolded proteins and activation of the unfolded-protein response (UPR) [78]. Upon UPR, protein kinase RNA-like endoplasmic reticulum kinase (PERK) is activated, phosphorylating CHK1 and Claspin to slow replication fork progression and reduce origin firing [79]. However, our data did not suggest differences in the accumulation of unfolded proteins (electronic supplementary material, figure S3E). Additionally, we did not detect the phosphorylation of CHK1 (figure 5*a*; electronic supplementary material, figure S4B), and replication fork speed and origin firing were unaffected by proteasome inhibition (electronic supplementary material, figure S4D,E). Therefore, the hypersensitivity to bortezomib in TKO cells is likely UPR-independent. By contrast, we observed elevated replication fork asymmetry upon bortezomib treatment in TKO cells (figure 4*i*), suggesting a higher propensity of fork stalling. Lastly, TKO cells had a significant increase in the NRS-phase population (figure 4*f*), suggesting that these cells are less likely to restart replication upon prolonged fork stalling. Together, these data suggest that the cooperation between RNF4 and USP7 is critical in sustaining replication progress and potential fork restart.

The DNA replication defects and subsequent increased aberrant anaphases (45%) in TKO cells treated with bortezomib (figure 4*g*) correlated with elevated apoptosis (41%) (figure 3*c*). Although it is unclear how cells induce p53-independent apoptosis in our model, there are several possible mechanisms. Chromosome mis-segregation due to incomplete replication or DNA damage induces prolonged mitotic arrest [80]. Mitotically arrested cells either complete division, often forming aneuploid cells, or die during mitosis [81]. Mitotic death can be caused by caspase activation and subsequent mitochondrial damage in a p53-independent manner [82]. Additionally, unprotected stalled replication forks (due to p53-deficiency) might release DNA into the cytoplasm [83], leading to activation of the cyclic GMP-AMP/synthase-stimulator of interferon genes (cGAS-STING) pathway [84] and subsequent caspase-dependent apoptosis [85,86]. Coincidently, it has been shown that bortezomib induces caspase-3-dependent cell apoptosis in p53-null cells [87]. It is likely that TKO cells treated with bortezomib undergo caspase-dependent apoptosis through one or both mechanisms. Interestingly, ATR/CHK1/p21 signalling suppresses caspase-dependent apoptosis following replication stress regardless of p53 status [88,89]. As a result, the lack of ATR activity upon bortezomib treatment (figure 5) might further enhance caspase-mediated apoptosis in TKO cells.

Bortezomib therapy is associated with severe adverse effects including peripheral neuropathy [90]. Additionally, both *de novo* and acquired resistance to bortezomib have limited the clinical use of bortezomib [62]. To address these limitations, scientists have attempted to combine bortezomib with other therapeutics, such as irradiation or cisplatin [64,91,92]. We found that proteasome and ATR activities are epistatic, as our data showed that bortezomib suppressed ATR-mediated RPA phosphorylation upon UV exposure. Furthermore, CHK1 was not phosphorylated and origin firing was unaffected despite DNA replication being severely dysregulated when cells were treated with bortezomib (figure 5; electronic supplementary material, figure S4E). Therefore, it might not be beneficial to administer proteasome inhibitors with an ATR inhibitor or CHK1 inhibitor. Bortezomib sensitizes tumour cells to DNA-damaging agents by lowering the levels of DSB repair factors in short-term *in vitro* experiments [64,93]. Nevertheless, long-term experiments have shown no sensitizing effect of bortezomib due to the high tumour heterogeneity in mouse models [92]. Furthermore, cancer cells with defective p53 are more resistant to bortezomib than p53-proficient cells due to the expression of the apoptotic pathway inhibitor survivin [94]. With our data showing that *RNF4* or *USP7* mutants exhibited hypersensitivity to proteasome inhibitors (figure 3*a–c*), we propose that a prescreen to identify patients carrying mutations in these ubiquitin-related genes might increase the efficacy of bortezomib therapy or overcome bortezomib resistance in p53-deficient patients.

## 4. Materials and methods

### 4.1. Cell culture and drug treatment

Human telomerase reverse transcriptase (hTERT)-immortalized RPE-1 cells were grown in DMEM/F12 medium (Thermo Fisher no. 11320033) supplemented with 10% FBS (Sigma no. 12306C) and 1% Pen/Strep (Gibco no. 15140-122) at 37°C and 5% CO_2_.

For bortezomib treatment, cells were seeded in plates and recovered for 24 h. Medium containing 6 nM (or otherwise indicated) of bortezomib (Millipore Sigma no. 504314) was added for 12, 24 or 48 h, as shown in different assays.

### 4.2. Cell lines generation using CRISPR/Cas9 and plasmid transfection

RPE-1 *RNF4^−/–^* cell lines were generated using CRISPR/Cas9 plasmid-based gene targeting. Single guide RNAs (sgRNAs; 5′-TGCTTCCAAGGAGATCTCGG-3′) were cloned into a CRISPR/Cas9 plasmid hSpCas9(BB)-2A-GFP (PX458, Addgene no. 48138) [95]. Early passage RPE-1 wild-type (WT) cells were transfected with CRISPR/Cas9 containing sgRNA using the Neon Transfection System (Invitrogen MPK5000 and MPK1096) following the manufacturer's protocol. Forty-eight post-transfection, GFP-positive cells were isolated by flow sorting and subcloned into 96-well plates. Subcloned cells were screened by PCR amplification followed by *Bgl*II restriction enzyme digestion.

RPE-1 *Cas9^O/E^/PuroR^−/–^/TP53^−/–^* cell lines were generated as previously described [49].

RPE-1 *RNF111^−/–^* cell lines were generated by transfecting the parental cells using the Neon Transfection System with 1 ng of chemically synthesized sgRNA (Synthego Corporation; 5′-TCAGGAGTCCATTGAGACAT-3′) and 1 µg of Cas9 mRNA (TriLink no. L-7206) following the manufacturer's protocol. Seventy-two hours post-transfection, cells were subcloned into 96-well plates. Subcloned cells were screened by PCR amplification followed by *Bco*DI restriction enzyme digestion.

RPE-1 *Cas9^O/E^/TP53^−/–^/USP7^−/–^* and RPE-1 *Cas9^O/E^/TP53^−/–^/RNF4^−/–^/USP7^−/–^* cell lines were generated by transfecting the parental cells using the Neon Transfection System with 1 ng of chemically synthesized sgRNAs (5′-GGTTCTGAGTAATTCTTGGT-3′) following the manufacturer's protocol. Seventy-two hours post-transfection, cells were subcloned into 96-well plates. Subcloned cells were screened by PCR amplification followed by TIDE analysis.

To complement RPE-1 *RNF4^−/–^* cell lines, we first cloned EGFP-tagged *RNF4* cDNA into pDONR201 (Invitrogen no. 11798-014) by Gateway BP cloning (Invitrogen no. 11789) and then cloned the cDNA into the expression vector PiggyBac Sleeping Beauty (PBSB) using the Gateway LR cloning (Invitrogen no. 11791) protocol. Next, parental cells were transfected with PiggyBac PBSB and PiggyBac-transposase plasmids by Lipofectamine 3000 (Thermo Fisher no. L3000-008) following the manufacturer's protocol. Stable cell lines were selected using 0.5 µg ml^−1^ of G418 (Millipore Sigma no. A1720). Subcloning was performed with flow cytometry isolating low EGFP expressing cells.

To complement RPE-1 *USP7^−/–^* cell lines, we transfected the parental cells with pcDNA3.1-N-Myc_WT USP7 (Addgene no. 131242) [96] via Lipofectamine 3000. Stable bulk populations were generated using 0.5 µg ml^−1^ of G418 selection.

### 4.3. Genetic screens with targeted CRISPR/Cas9 library

The genetic screens were performed as previously described [49]. The custom DNA damage repair (DDR) sgRNA library was synthesized (Cellecta) and packaged into a lentiviral library. RPE-1 *Cas9^O/E^/PuroR^−/–^/TP53^−/–^* and RPE-1 *Cas9^O/E^/PuroR^−/–^/TP53^−/–^/RNF4^−/–^* cells were seeded in 15 cm plates to ensure more than 1000X representation of the sgRNA library and allowed to recover for 24 h. Cells were transduced with the sgRNA lentiviral library at an MOI of 0.2. Twenty-four hours post-transduction, cells were selected with 3 µg ml^−1^ of puromycin (Millipore Sigma no. P8833) until the un-transduced control cells were completely dead. The puromycin-resistant sgRNA library cells were then split into triplicate and propagated every 3 days. Cells were collected every 3 days from day 0 (T0) to 18 (T18). Note that cells were kept as independent technical replicates (i.e. cells were not pooled together after T0) throughout the screen.

Genomic DNA was extracted from all cell pellets using the Wizard Genomic DNA Purification Kit (Promega no. A1120) following the manufacturer's instructions. Two-round PCR was performed using the NGS prep kit for sgRNA libraries in pRSG16/17 (KOHGW) (Cellecta no. LNGS-120) and the Supplementary Primer Sets (Cellecta no. LNGS-120-SP) following the manufacturer's instructions with modifications. Twenty micrograms of genomic DNA (the equivalent of 1000X coverage of the custom DDR sgRNA library) was used in the two-round PCR, which enriched the sgRNA from the genomic DNA, reduced genomic DNA carryover and allowed for multiplex identifiers barcodes to be added into different samples. PCR products were purified with a PCR purification kit (Qiagen no. 28104) and a gel-extraction kit (Qiagen no. 28704). Purified PCR products were sequenced on Illumina NextSeq 550 (standard Single-Read (SR) 150-cycles).

### 4.4. Cell proliferation assay

Cells were plated at 100 000 cells per well in six-well plates. Two days after seeding, cells were washed with 1X PBS and fresh medium was added. After 24 h, cells were counted using Trypan Blue (Invitrogen no. T10282) on Countess slides (Invitrogen no. C10283) using a Countess II Automated Cell Counter (Invitrogen no. AMQAX1000).

### 4.5. ATPase cell viability assay

Cells were seeded at 500–1000 cells per well in quadruplicate in 96-well plates and allowed to recover for 24 h. Stock solutions of each compound were prepared in sterile dimethyl sulfoxide (DMSO) or water. Serial dilution of stock solutions in a growth medium was performed when preparing each drug concentration. Cells were then incubated in a drug-containing medium for 96 h. Cell viability was measured using the CellTiterGlo luminescent assay (Promega no. G75752) according to the manufacturer's instructions. Luminescence intensities were measured on a Promega GloMax Microplate Reader. The relative survival of drug-treated versus untreated cells was expressed as percentage of the untreated control. Dose–response curves and IC_50_ values were analysed using GraphPad Prism9 with an asymmetrical (five-parameter) logistic dose–response model.

### 4.6. Whole cell extract preparation and chromatin fractionation

For preparation of whole cell extracts, cells were lysed in RIPA (50 mM Tris–HCl pH 8.0, 150 mM NaCl, 10 mM NaF, 1% NP-40, 0.1% SDS, 0.4 mM EDTA, 0.5% sodium deoxycholate and 10% glycerol) buffer on ice for 10 min and then centrifuged at 16 000×g for 10 min. Cleared lysates were collected and protein concentrations were determined by Protein Assay Dye Reagent (Bio-Rad no. 5000006).

For chromatin fractionation, cells were lysed in buffer A (10 mM HEPES pH 7.9, 10 mM KCl, 1.5 mM MgCl_2_, 0.34 M sucrose, 10% glycerol, 0.1% Triton X-100 and protease/phosphatase inhibitors) on ice for 5 min and then centrifuged at 1300×g for 10 min at 4°C. Pellets (chromatin-bound proteins) were then resuspended in TSE buffer (20 mM Tris–HCl pH 8.0, 500 mM NaCl, 2 mM EDTA, 0.1% SDS, 1% Triton X-100 and protease/phosphatase inhibitors) followed by sonication and centrifugation at 17 000×g for 10 min at 4°C. Clear lysates were collected and protein concentrations were determined by Protein Assay Dye Reagent as described previously [97].

### 4.7. Western blotting

Protein lysates were mixed with 4× Laemmli protein sample buffer (Bio-Rad no. 1610747) containing 200 mM DTT (Millipore Sigma no. D0632) and boiled for 10 min. Protein samples were resolved on 4–15% Mini-PROTEAN TGX precast protein gels (Bio-Rad no. 4561084 and no. 4561086) and transferred onto a nitrocellulose membrane (Bio-Rad no. 1620115). The membrane was blocked in 5% BLOT-QuickBlocker (G-Bioscience no. 786-011) in TBS-T (20 mM Tris–HCl, 150 mM NaCl and 0.1% (w/v) Tween 20) at room temperature (RT) for 1 h and incubated with primary antibodies at 4°C overnight. Membranes were washed with TBS-T three times (10 min each) and incubated with secondary antibodies at RT for 1 h. Membranes were washed with TBS-T three times (10 min each) and detection was performed using WesternBright Quantum detection kit (Advansta no. K-12042-D20) and HyBlot ES high sensitivity autoradiography film (Thomas Scientific no. E3212).

Primary antibodies were prepared in 5% BLOT-QuickBlocker in TBS-T as follows: goat anti-RNF4 (R&D Systems no. AF7964, 1 : 500), rabbit anti-USP7 (Abcam no. ab4080, 1 : 2000), mouse anti-p53 (Santa Cruz no. sc-126, 1 : 1000), mouse anti-Ku86 (Santa Cruz no. sc-5280, 1 : 5000), rabbit anti-pS345-CHK1 (Cell Signaling no. 2348S, 1 : 500), mouse anti-CHK1 (Cell Signaling no. 2360, 1 : 1000), rabbit anti-pS33-RPA32 (Bethyl Laboratory no. A300-246A, 1 : 1000), mouse anti-RPA32 (Abcam no. ab2175, 1 : 500), rabbit anti-FANCD2 (Abcam no. ab108928, 1 : 2000), rabbit anti-RAD51 (Santa Cruz no. sc-8349, 1 : 1000), rabbit anti-γH2AX (Bethyl Laboratory no. A300-081A, 1 : 2000), mouse anti-Flag M2 (MilliporeSigma no. F3165, 1 : 1000), rabbit anti-GFP (Abcam no. ab290, 1 : 2000), rabbit anti-lamin B1 (Proteintech no. 12987-1-AP, 1 : 3000), and rabbit anti-SUMO2/3 (Millipore no. 07-2617, 1 : 1000). Secondary antibodies were prepared in 5% BLOT-QuickBlocker in TBS-T as follows: rabbit anti-goat HRP conjugate (R&D systems no. HAF017, 1 : 2000), goat anti-mouse HRP conjugate (Jackson Laboratories no. 115-035-003, 1 : 10 000), and goat anti-rabbit HRP conjugate (Jackson Laboratories no. 111-035-003, 1 : 10 000).

### 4.8. Chromatin flow analysis

DNA synthesis, cell cycle, and chromatin-bound proteins were analysed by flow cytometry as previously described [97]. Cells were seeded in 6 cm plates and treated with drugs. Before cells were harvested, cells were incubated with 10 µM EdU (Lumiprobe no. 10540) for 30 min at 37°C. Cells were then harvested, washed with cold 1X PBS, and soluble proteins were extracted in cold CSK (10 mM PIPES pH 7.0, 300 mM sucrose, 100 mM NaCl, 3 mM MgCl_2_, 0.5% Triton X-100 and protease/phosphatase inhibitors) for 10 min. Cells were then fixed with 4% PFA (Electron Microscopy Services, no. 15714) in PBS for 15 min at RT. To detect chromatin-bound proteins, we incubated the cells with primary antibodies for 1 h at 37°C, washed them with 1% bovine albumin serum (BSA) in PBS with 0.1% NP-40, and incubated them with secondary antibodies for 1 h at 37°C. Cells were incubated in EdU detection solution (1 µM AF647-azide (Life Technologies no. A10277), 100 mM ascorbic acid, 1 mM CuSO4 in PBS) for 30 min at RT for EdU detection. Lastly, cells were washed and incubated in 1 µg ml^−1^ of DAPI (Life Technology no. D1306) and 100 µg ml^−1^ RNaseA (Sigma no. R5125) in 1% BSA in PBS with 0.1% NP-40 overnight at 4°C. Samples were run on LSR II or LSRFortessa (BD Biosciences) flow cytometer and analysed with FlowJo v.10.6.1.

Primary antibodies were prepared in 1% BSA in PBS with 0.1% NP-40 as follows: rabbit anti-pS33-RPA32 (Bethyl Laboratory no. A300-246A, 1 : 400), muse anti-RPA32 (Abcam no. ab2175, 1 : 500) and rabbit anti-γH2AX (Cell Signaling no. 9718S, 1 : 500). Secondary antibodies were prepared in 1% BSA in PBS with 0.1% NP-40 as follows: goat anti-mouse Alexa Fluor 488 (Invitrogen no. A11029, 1 : 1000), donkey anti-rabbit Alexa Fluor 488 (Invitrogen no. A21206, 1 : 1000), goat anti-mouse Alexa Fluor 594 (Invitrogen no. A11032, 1 : 1000) and goat anti-rabbit Alexa Fluor 594 (Invitrogen no. A31632, 1 : 1000).

### 4.9. PROTEOSTAT aggresome detection assay

Unfolded proteins were detected using the PROTEOSTAT aggresome detection assay (Enzo Life Science no. ENZ-51035) according to the manufacturer's instructions. In brief, cells were seeded at 300 000 cells per well in 6-well plates and allowed to recover for 24 h. Cells were treated with or without 6 nM of bortezomib or 2 µM of MG132 for 24 h. Cells were collected and washed with PBS once and resuspended in a small volume of PBS. Cells were then fixed by adding 4% formaldehyde solution dropwise into the cell suspension and incubated at RT for 30 min. Fixed cells were collected by centrifugation, washed with PBS, and resuspended in a small volume of PBS. Permeabilizing solution (0.5% Triton X-100 and 3 µM EDTA pH 8.0 in 1X Assay Buffer) was added dropwise into the cell suspension and cells were incubated on ice for 30 min. Cells were then collected by centrifugation, washed with PBS, resuspended in a small volume of PBS and transferred to FACS tubes with a cell strainer cap (Corning no. 352235). Cells were centrifuged, resuspended in freshly diluted PROTEOSTAT Aggresome Red Detection Reagent and incubated at RT for 30 min in the dark. Samples were analysed on a BD LSR II flow cytometer with FlowJo v.10.6.1.

### 4.10. Cell apoptosis assay

Cells were plated at 100 000 cells per well in six-well plates and allowed to recover for 24 h. Cells were treated with or without 6 nM of bortezomib for 48 h. Both adherent and floating cells were collected and washed with cold cell staining buffer (BioLegend no. 420201) twice and stained using the APC Annexin V apoptosis detection kit (BioLegend no. 640932) according to the manufacturer's instructions. Samples were analysed on a BD LSR II flow cytometer with FlowJo v.10.6.1.

### 4.11. DNA combing analysis

For genome-wide analyses of DNA replication, cells were plated at 40% confluency in 15 cm plates 24 h prior to labelling. Cells were incubated with 25 µM of IdU (Sigma no. C6891) for 30 min, rinsed twice with a pre-warmed medium and then incubated with 200 µM of CldU (Sigma no. I7125) for 30 min. Approximately 250 000 cells were embedded in 0.5% agarose plugs (NuSieve GTG Agarose, Lonza, 50080) and digested for 72 h in plug digestion solution (10 mM Tris–HCl, pH 7.5, 1% Sarkosyl, 50 mM EDTA and 2 mg ml^−1^ Proteinase K (Macherey-Nagel no. 740506)). Plugs were melted in 50 mM MES pH 5.7 (Calbiochem no. 475893) and digested overnight with β-agarase (NEB no. M0392). DNA was subsequently combed onto commercially available vinyl silane-coated coverslips (Genomic Vision no. COV-001). The integrity of combed DNA for all samples was checked by staining with YOYO-1 dye (Invitrogen no. Y3601). Combed coverslips were baked at 60°C for 2–4 h, cooled to RT and stored at −20°C. DNA was denatured in 0.5 M NaOH and 1 M NaCl for 8 min at RT. All antibody staining was performed in 2% BSA in PBS with 0.1% Triton X-100. Primary antibodies included rabbit anti-ssDNA (IBL no. 18731), mouse anti-BrdU/IdU (BD Biosciences no. 347580; clone B44) and rat anti-BrdU/CldU (Abcam no. ab6326; BU1/75 (ICR1)). Secondary antibodies included goat anti-mouse Cy3.5 (Abcam no. ab6946), goat anti-rat Cy5 (Abcam no. ab6565) and goat anti-rabbit BV480 (BD Horizon no. 564879). Imaging was performed using Genomic Vision EasyScan service. Images were blinded and analysed using the Genomic Vision FiberStudio software. Data/statistical analyses were performed in GraphPad Prism 9 as described previously [97].

### 4.12. Anaphase analysis

Cells were seeded at 100 000–200 000 cells per sterilized coverslips in six-well plates and allowed to recover for 24 h. Cells were treated with or without 6 nM of bortezomib for 24 h. Cells were washed with PBS and fixed with cold formalin for 15 min and incubated with DAPI for 10 min. Slides were allowed to dry and were mounted using the Vectashield mounting medium (Vector Laboratories H1000); 50–100 anaphases per condition were scored per experiment using the EVOS FL imaging system (ThermoFisher AMF43000).

### 4.13. 53BP1 immunofluorescence staining and image analysis

Cells were seeded 100 000 or 200 000 cells per sterilized coverslips in six-well plates and allowed to recover for 24 h. Cells were fixed with 3.7% formaldehyde (Fisher Scientific F79-500) in PBS for 10 min at RT, washed twice with PBS and permeabilized with 0.1% Triton X-100 in PBS for 5 min, followed by two washes with PBS. Cells were blocked in ABDIL (20 mM Tris pH 7.5, 2% BSA, 0.2% fish gelatine, 150 mM NaCl, 0.1% sodium azide) at RT for 1 h. Cells were incubated in the primary antibody solution overnight at 4°C in a humid chamber. Coverslips were washed three times with PBS-T (0.1% Tween-20 in PBS) and incubated in the secondary antibody solution at RT for 1 h. Coverslips were washed three times with PBS-T and 1 µg ml^−1^ of DAPI was added to the PBS-T during the second wash. Coverslips were mounted using the Vectashield mounting medium. Coverslips were inspected under a Zeiss Spinning Disk confocal fluorescent microscope. Images were scored using FIJI and the number of 53BP1 NBs was counted specifically in G1 cells, which were identified as cyclin A-negative cells.

Primary antibodies were prepared in ABDIL as follows: rabbit anti-53BP1 (Abcam no. ab36823, 1 : 500) and mouse anti-cyclin A (Santa Cruz no. sc-271682, 1 : 200). Secondary antibodies were prepared in ABDIL as follows: donkey anti-rabbit Alexa Fluor 488 (Invitrogen no. A21206, 1 : 1000) and goat anti-mouse Alexa Fluor 594 (Invitrogen no. A11032, 1 : 1000).

### 4.14. Data analysis for genetic screen data

#### 4.14.1. Converting fastq files to raw read counts

Demultiplexed FASTQ files were generated using the Illumina bcl2fastq software. These files were used as input for the Cellecta ‘NGS Demultiplexing and Alignment Software’, along with a ‘Sample Description File’ that matches index barcode to sample and a ‘Library Configuration File’ containing a list of target sgRNA guide sequences. The Cellecta software generated a table of raw read counts for each sgRNA (row) and each sample (column).

#### 4.14.2. Genetic interaction scoring

GIs were scored using an adapted version of the Orthrus software [52]. Raw read counts were normalized by read depth for each sample. Guides with low (less than 30) or high (greater than 10 000) levels of raw read counts were excluded from the analysis. Per-guide-level log_2_ fold changes (LFCs) were calculated between an intermediate or end timepoint (T18) and starting timepoint (T0). LFC values underwent two additional normalization steps: (i) MA-transformation, where guide-level LFC differences between mutant and control conditions (M) were plotted against guide-level LFC averages of mutant and control (A) and (ii) loess (locally estimated scatterplot smoothing) regression, which bins the data with equal bin sizes along the A values and fits a smooth curve through the data points within each bin. Replicate normalized LFC values are averaged before downstream steps. Then, the guide-level GI scores were derived from calculating the differential normalized LFC values between *TP53^−/–^/RNF4^−/–^* DKO and *TP53^−/–^* screens. Guide-level GI scores were tested for gene-level significance using the moderated *t*-test from the *limma* R package [98] and then averaged to compute gene-level GI scores. *p*-values were FDR-corrected using Benjamini–Hochberg multiple testing correction.

#### 4.14.3. CRISPR screen quality control plots

To validate the CRISPR screen approach, we generated two quality control plots using Orthrus: (i) replicate correlation and (ii) essential gene dropout. Replicate correlation was computed with a Pearson's correlation coefficient on the vector of LFC values between all possible replicate pairs (AB, AC, BC). Using an essential gene standard defined by the Broad Dependency Map (DepMap) data (CERES score < −1 in greater than 60% of DepMap cell lines), we generated AUC-ROC (area under the ROC curve) values based on the LFC values for each screen to quantify how well essential genes drop out as expected throughout the length of the screen.

#### 4.14.4. GO-biological process-level interaction score analysis

Gene Ontology:Biological Process (GO:BP) analysis was performed on the GIs as follows. Mappings from GO:BP term to genes were downloaded from the ‘topGO’ R package. GO:BP terms with fewer than 3 library genes were filtered out, resulting in 1186 GO:BP terms for downstream analysis. A *z*-score was computed for each GO:BP term to summarize the direction and strength of GIs for genes within that term using the following formula:

$$Z=\frac{\bar{x}-\mu }{\sigma /\surd n},$$

where $\bar{x}$ = average GI score of all genes annotated to the GO:BP term, *μ* = the average GI score across all library genes, *σ* = the standard deviation of GI scores across all library genes, and *n* = number of genes annotated to the GO:BP term. A two-tailed *p*-value was calculated for each *z*-score using the R pnorm function. The *p*-values were then adjusted by the Benjamini–Hochberg method for multiple comparisons.

### 4.15. Statistical analysis

All the statistical analyses were performed in GraphPad Prism 9 or R. Statistical methods, descriptive statistics, and significance were indicated in each applicable figure legend.

## Data Availability

All data needed to evaluate the conclusions in the paper are present in the paper and the electronic supplementary material [99]. The DDR-focused targeted sgRNA library (3033 sgRNAs targeting 350 DNA damage response genes) is available from the corresponding author upon request. The datasets supporting the CRISPR/Cas9 genetic screens are available at Sequence Read Archive (SRA) database with the accession number PRJNA922953 (https://www.ncbi.nlm.nih.gov/sra/PRJNA922953).
